# Utilization of plasma as an ameliorator for forage productivity and in vitro traits of cowpea cultivated in salty soil

**DOI:** 10.1038/s41598-025-05498-9

**Published:** 2025-06-27

**Authors:** Hani S. Saudy, Mohamed F. Hamed, Taia A. Abd El-Mageed, Nasr E. El-Bordeny, Marwa A. Madkour, Mohamed H. Shokry, Gouda F. Gouda, Mariusz Jaremko, Abdul-Hamid Emwas, Abdelfattah T. Elgendy

**Affiliations:** 1 Agronomy Department, Faculty of Agriculture, Ain Shams University, Hadayek Shoubra, P.O. Box 68, Cairo, 11241 Egypt; 2 Soil and Water Department, Faculty of Agriculture, Fayoum University, Fayoum, 63514 Egypt; 3 Animal Production Department, Faculty of Agriculture, Ain-Shams University, Cairo, Egypt; 4 Animal Nutrition Department, Animal Production Research Institute, Agriculture Research Center, Dokki, Giza Egypt; 5 Agricultural Environmental Science Department, Faculty of Graduate Studies and Environmental Research, Ain-Shams University, Cairo, Egypt; 6 Department of Animal and Fish Production, College of Agricultural and Food Sciences, King Faisal University, Al-Ahsa, Saudi Arabia; 7 Division of Biological and Environmental Sciences and Engineering (BESE), Smart-Health Initiative (SHI) and Red Sea Research Center (RSRC), King Abdullah University of Science and Technology (KAUST), Thuwal, Jeddah Kingdom of Saudi Arabia; 8 Core Labs, King Abdullah University of Science and Technology (KAUST), 23955-6900 Thuwal, Kingdom of Saudi Arabia; 9 Physics Department, Faculty of Science, Ain Shams University, Cairo, Egypt

**Keywords:** Animal nutrition, Cold plasma, Cowpea yield, Forage quality, In vitro study, Soil salinization, Plant stress responses, Biological techniques, Animal behaviour

## Abstract

The adoption of advanced and practical technologies to boost plant productivity and improve quality under challenging environmental conditions, such as salinity, has become an essential need in modern agriculture. Plasma technology can significantly improve the seed’s resistance to stress factors like high salinity and dry environments. Thus, the current work aimed to improve the yield and quality of cowpea as an important forage crop grown in saline soil using a plasma coating approach. The seeds of cowpea were treated with three plasma doses expressed in different times of exposure (0.0, 1.0 and 2.0 min) and planted (for two seasons of 2022 and 2023) in three soil salinity levels expressed in electrical conductivity, EC (normal, 0.3 dS m^−1^, moderate salinity 5.5 dS m^−1^, and high salinity, 7.0 dS m^−1^, abbreviated as EC3.0, EC5.5 and EC7.0, respectively). The electron micrographs and elemental detection revealed that 2.0 min treatment resulted in deep cracking and topographical modulation with the best enhancements in cowpea seed surface nutrients. The agronomic findings revealed that compared to the corresponding check treatment (without plasma, 0.0 min), the exposure to plasma for 2.0 min in the first season was the efficient for enhancing forage yield under normal (1.37-fold increase) and medium salinity (1.79-fold increase). The in vitro data showed plasma-treated seeds for 2.0 min displayed higher acid detergent fiber content under EC3.0 or EC5.5 compared to the other treatments. Plants grown from seeds treated with plasma for 1.0 min showed higher dry matter degradability levels at EC7.0 compared to the other treatments. At EC7.0 the highest ammonia concentration was recorded in plants grown plasma-treated seeds for 1.0 min, while the lowest value was observed in 2.0-min. 2.0-min plasma-treated seeds produced the highest total volatile fatty acids across different salinity conditions, particularly at EC7.0. Plasma treatment, as a safe and innovative seed priming method, validates substantial potential in improving cowpea productivity under saline conditions. This study revealed that exposing cowpea seeds to a 2-min plasma treatment before sowing enhanced seed germination rate, and overall yield, even under challenging saline environments. Moreover, enhanced feed quality resulting from plasma-treated seeds offers direct benefits to livestock nutrition, supporting both human and animal food chains.

## Introduction

The need for advanced technologies to enhance plant productivity and quality under extreme environmental conditions, such as high salinity and drought, has become an urgent priority in agriculture^1–4^. As climate change continues affecting global weather patterns, agricultural systems worldwide face increasingly harsh challenges. Traditional farming practices are often insufficient to cope with these challenges, making exploring innovative solutions essential to ensure food security and sustainable agricultural practices^5,6^.

One such promising solution is the application of plasma technology, which has garnered significant attention for its potential to improve agricultural output. Plasma applications involves the use of non-thermal plasma (NTP) which exploits the ionized gases (plasma) to treat seeds, creating a unique, reactive surface that can alter the seed’s properties and enhance its ability to withstand stressors such as salinity and water scarcity. This process involves exposing seeds to low-temperature plasma, which results in a thin coating that can help improve seed germination, promote healthier growth, and increase tolerance to adverse conditions like saline soil and dry environments^7–11^. In addition, NTP covers several discharge techniques, such as dielectric barrier discharge (DBD), glow discharge, and atmospheric pressure plasma jets, each with distinct plasma chemistry and energy profiles. For instance, DBD-based plasma systems have shown significant promise in stimulating seedling growth and mitigating environmental stress by enhancing seed surface hydrophilicity and activating beneficial metabolic pathways^12,13^. In addition, Diffuse Coplanar Surface Barrier Discharge (DCSBD) plasma has proven effective in promoting seed germination in forest species by modifying surface morphology and increasing water uptake^14^. Glow discharge plasma, typically applied under low pressure, has also been explored for its efficacy in improving metabolic activation and energy conversion processes during early germination phases^15^. Both atmospheric and low-pressure plasma systems have demonstrated the ability to tailor reactive oxygen and nitrogen species (RONS), which act as bio-stimulants for seed priming, leading to enhanced germination rates, improved root-shoot development, and increased tolerance to abiotic stressors^16,17^.

The agricultural sectors in these situations encounter serious ecological problems such as unfavorable temperatures, low fertile soils, drought, and salinization^18–22^. When plants are exposed to such stressors, dramatic effects occur, causing problems in physiological conditions and growth^23–26^. In this context, salinity is one of the critical stressors for plant growth, as elevated salts in soil can harm the plants via physiological dehydration and toxicity^27–29^. Accordingly, crop plants that are cultivated in salty soils should be safeguarded by substantial practices to ensure healthy growth and development^30,31^. Herein, unconventional and smart agricultural tactics should be adopted to obtain acceptable yield and quality under mischievous environments^32–34^.

NTP as an emerging tactics can be generated at low pressure and temperature^9,10^. There are limited researches that have studied the potentiality of NTP for affecting the morphological and biochemical traits of seeds and resilience to metal toxicity and drought^35^, demonstrating that NTP treatment could enhance seed characteristics. Studies have shown the effectiveness of plasma in boosting oxidative stress tolerance, nutrient absorption, and water utilization under challenging conditions^7^.

The application of plasma technology in agriculture represents a cutting-edge development with the potential to significantly transform farming practices. By integrating plasma treatments into seed production and crop management, farmers can improve their resilience to environmental stresses, increase crop yields, and ensure more sustainable farming in regions facing adverse conditions. As research into plasma technology continues to advance, it holds great promise for revolutionizing agricultural practices, improving food security, and ensuring the long-term sustainability of global agriculture in the face of climate change. However, further research is needed to optimize the parameters of plasma treatment, including exposure time, intensity, and frequency, to maximize its benefits for various crops and under different environmental conditions. Future studies could explore the long-term effects of plasma-treated seeds on plant health, soil quality, and ecosystem sustainability. As technology advances, it could pave the way for more resilient and sustainable agricultural practices that can help address the challenges posed by climate change and soil degradation. Ultimately, plasma technology has the potential to transform farming practices, ensuring better yields, improved crop quality, and greater resilience in the face of environmental stressors.

Despite the seed treatment by plasma possessing hopeful findings in germination and seedling growth^36,37^, rare knowledge is available about the effect of NTP on yield performance and product quality. Therefore, the current work hypothesized that treating cowpea seeds with NTP before sowing in saline soils could secure better plant establishment, growth, and yield. Additionally, NTP may modulate biomass quality by improving the in vitro traits. Accordingly, this study aims to evaluate the effects of NTP treatment on cowpea growth, and stress tolerance under saline conditions. Additionally, we explore the potential of plasma-treated cowpea as high-quality forage for ruminants, assessing its chemical composition, degradability, and fermentation properties. Herein, the NTP-treated seeds were sown in soil with different salinity degrees to assess productivity and forage quality.

## Materials and methods

### Plasma specification

A low-pressure capacitively coupled non-thermal plasma (CCP-NTP) reactor was used for seed surface modification. The discharge was generated using a 13.56 MHz RF power supply (up to 100 W), applied between two parallel circular stainless-steel electrodes inside a vacuum chamber. The plasma discharge conditions included a pressure of 1 mbar, a gas temperature of 300 K, an applied voltage of 250 V, and a power range of 0–100 W. The ion current flux (*eψ*_*i*_) is an important parameter in plasma physics and surface modification processes, as it quantifies the flux of ions hitting the surface (in our case study, cowpea seeds). Let’s find its value, importance, and role in ensuring effective surface modification.

The control group of cowpea seeds remained untreated (0.0 min), while the plasma treatment groups (1.0 and 2.0 min) received a specialized seed coating composed of lignin, activated charcoal, and hash carbon. This homogeneous lignocellulosic biofilm formed a protective extracellular matrix. To ensure adhesion and moisture resistance, the coated seeds were air-dried for a full day. Following coating, seeds were subjected to NTP exposure for 1.0 min or 2.0 min.

#### Evaluation of ion current flux (eψ_i_)

To estimate the ion current flux (eψ_i_) involved in the plasma-seed interaction, a macroscopic, power-based approach was employed. This method provides a practical approximation using the relationship between applied power (P), voltage (V), and total current (I), allowing researchers to estimate ion flux without relying solely on microscopic diagnostics. The total power delivered to the plasma system is given by the following standard Eqs. (1–4):

1 $$P = V \cdot I,$$

where P = power (W), V = applied voltage (V), I = total current (A), The ion current flux (e$\psi_{i}$) is given by formula (3).

2 $$\psi_{i} = \frac{I}{A},$$

where A = area of the plasma-treated surface (m^2^), Using the provided data: Applied voltage (V) = 250 V, Power (P) = 100 W (maximum value), Assumed seed surface area: A = 0.01 m^2^.

3 $$I = \frac{P}{V} = \frac{{100\,{\text{W}}}}{{250\,{\text{V}}}} = 0.4\,{\text{A}},$$

4 $$e\psi_{i} = \frac{{0.4\,{\text{A}}}}{{0.01\,{\text{m}}^{2} }} = 40\,{\text{A/m}}^{2} = 40\,{\text{mA/m}}^{2} .$$

This macroscopic model links measurable plasma electrical parameters to seed surface charge dynamics, bridging plasma physics and seed biology to enable predictive modeling of plasma-seed interactions across diverse complex plasma systems.

#### Mathematical model for plasma treatment

A suitable mathematical model describes the relationship between plasma parameters (e.g., power, time, ion current flux) and the biological response (e.g., germination rate, growth parameters). A dose–response model that can be used is presented as follows in Eq. (5).

5 $$Y = Y_{{{{\max}}}} \left( {1 - e^{ - kD} } \right),$$

where Y = biological response (e.g., germination rate, seedling growth), $Y_{{{{\max}}}}$ = maximum achievable response, k = efficiency constant, D = plasma dose.

In this study, plasma dose (*D*) is defined as the total ion flux delivered to the seed surface during treatment, calculated via Eq. (6).

6 $$D = e{\uppsi }_{i} \cdot t,$$

This definition was chosen because the ion flux is the primary driver of surface modifications, such as the introduction of functional groups and the formation of a protective layer, which enhances seed performance in saline soil. Plasma dose (*D*) can be computed by Eq. (7).

7 $$D = 40\,{\text{mA/m}}^{2} \times {{\min}}$$

**Fig. 1:**
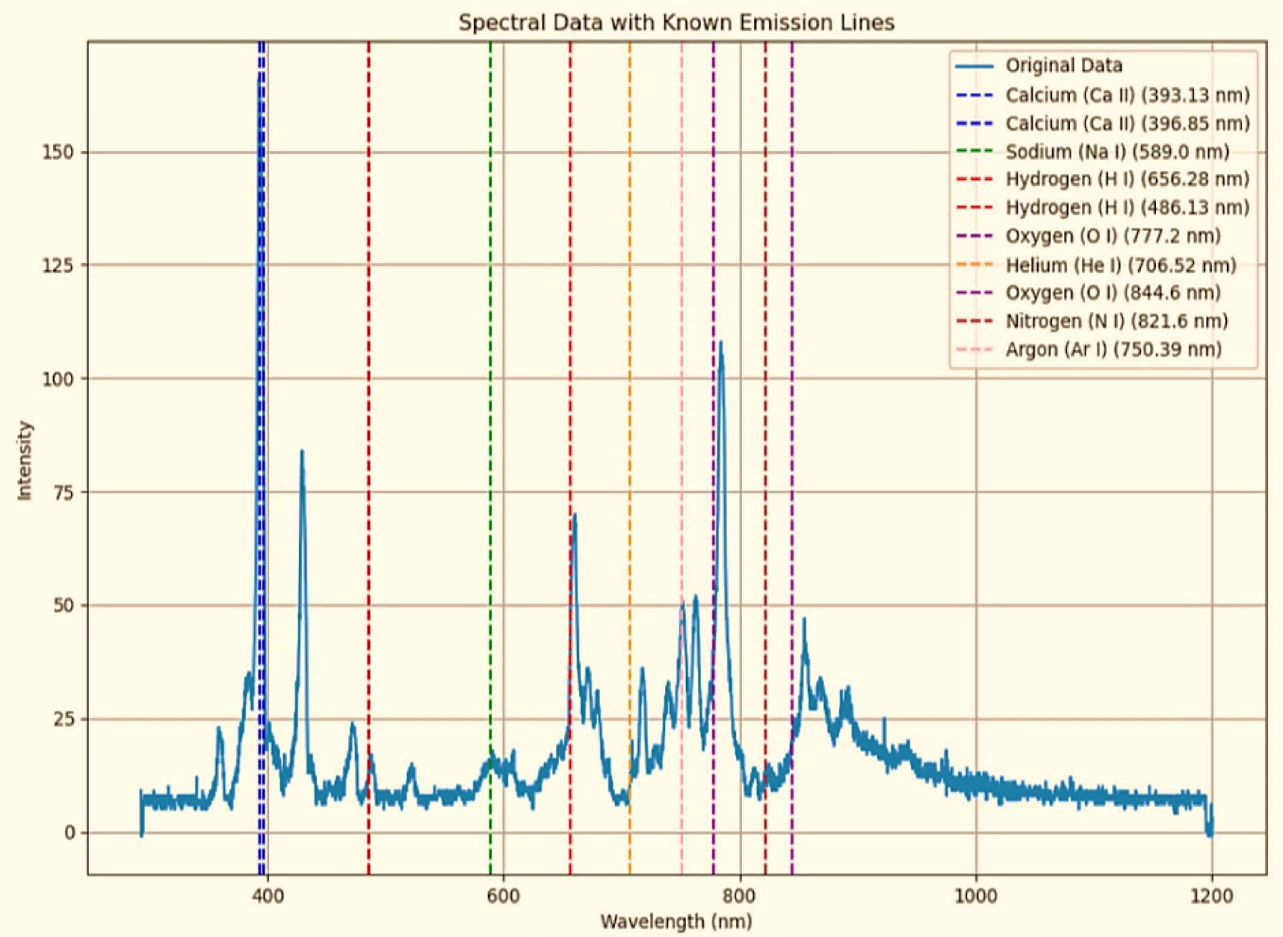
The spectral data indicate the presence of hydrogen (H), oxygen (O), and calcium (Ca) in the plasma sample. There is also potential evidence of helium (He) and sodium (Na). However, there is no clear evidence of nitrogen (N) or argon (Ar) in the sample.

This model can be fitted to experimental data to quantify the effect of plasma treatment. Figure 1 highlights key spectral peaks at 777 nm (O), 868 nm (N), 656 nm, and 430 nm (C), indicating the presence of oxygen, nitrogen, hydrogen, and carbon species in the plasma, which are critical for modifying the cowpea seed surface structure and enhancing its functional properties. Table 1 illustrates the summary of ion current flux value and importance.

**Table 1:** Summary of surface modifications of cowpea seeds before and after plasma treatment.

Parameter	Untreated (control)	Plasma-treated seed	Plasma + lignin treated seed	Unit
Primary components	Cellulose, lignin, proteins, lipids	Base components modified	Coating (lignin, activated charcoal)	–
Surface functional groups	–OH, –CH_3_, –C=O	–OH, –COOH, –NOx	–OH, –COOH, –NOx, –C=O	–
Surface energy	Low to moderate	High	Very high	mJ/m^2^
Surface roughness	Smooth	Increased (micro/nanoscale)	Further increased (coating fills roughness)	nm (nanometers)
Pathogen adhesion	High	Reduced	Very low	–
Coating adhesion	N/A	N/A	Strong (hydrogen bonding)	–
Ion current flux ($e{\uppsi }_{i}$)	N/A	(0–40.0) mA/m^2^	(0–40.0) mA/m	Controls surface modification intensity
Plasma dose (D)	N/A	(0–80.0) mA/m^2^ Min	(0–80.0) mA/m^2^·Min	Enhances hydrophilicity, germination
Biological correlation	N/A	+ (Enhanced hydrophilicity, salinity tolerance)	++ (Nutrient enrichment, protective layers)	–

### Seed treatment by plasma

In this study, cowpea seeds (obtained from Forage Crops Research Department, Agricultural Research Centre, Giza, Egypt) were treated with NTP using an alternating current (AC) plasma system operating at 13.56 MHz for 1.0 or 2.0 min. The plasma treatment occurred under a low vacuum (1 mbar) with air gas as the working medium, generating reactive species such as nitrogen, oxygen, carbon, and OH groups. These species chemically modified the seed surface, enhancing surface energy and improving interactions with coating materials.

### Scanning electron microscopy (SEM)

The morphology of seed surface was analyzed using a Quattro SEM (ThermoFisher Scientific) supplied with a field emission gun (FEG). For capturing the images, seeds were sputter-coated, and scanning electron microscopy (SEM) was utilized at an accelerating voltage of 5.0 kV using a secondary electron detector.

### Agronomic study

To outstanding the most appropriate plasma exposure time (dose) to be applied under open field conditions, a pilot experiment was conducted in wire house. In this regard, seeds of cowpea i.e. Sids-19 received three plasma doses expressed as time of exposure by minute, min (1.0, 2.0 and 3.0 min), in addition to the check treatment (non-plasma treated seeds, 0.0 min). On 25th May 2022, in a wire house, seeds were sown in plastic griddles (4.5 cm × 4.5 cm × 6.0 cm), containing 40 holes filled with soil, four seeds per hole were sown. At 14 days after sowing (DAS), the excess seedlings were removed to leave two seedlings per hole. For determining the most efficient plasma doses for cowpea growth based on the behavior of seedling growth and development, relative chlorophyll content expressed in SPAD reading was assessed using a chlorophyll meter, SPAD–502, KONICAMINOLTA. Inc., Tokyo) at 20 DAS, Further, seedling length and seedling fresh weight were estimated.

#### Agronomic procedures

To assess the potentiality of plasma in alleviating the adverse impacts of soil salinity, open field trials were carried out in the 2022 and 2023 summer seasons at Fayoum Governorate, Egypt (29° 18′ N and 30° 56′ E), representing a model of salt-affected soils area. According to the aridity index^38^, the study location is typified by an arid climate. At 0–30 cm soil depth, samples were obtained before sowing to evaluate the soil profile, including physical^39^ and chemical^40^ properties. The soil was a sandy loam with 1.71 g cm^−3^ bulk density, 41.0% total porosity, 12.2% water-holding pores, and 2.21 cm h^−1^ hydraulic conductivity. Additionally, the soil had 0.62% organic matter, 4.40% calcium carbonate, and a pH of 7.9.

Soil was ploughed two times while incorporating single superphosphate (15.5% P_2_O_5_) at arte of 70 kg P_2_O_5_ ha^−1^. Then, the experimental land was sectioned into plots (9.0 m^2^) with 5.0 rows, 3.0 m length and 0.60 m apart. Based on the promising findings obtained from the wire house experiment the plasma dose of 3.0 min was excluded, since it caused exfoliation and carbonization dominate the seed surface and its effect on seedling growth was as similar as the plasma dose of 2.0 min. Accordingly, before sowing, cowpea seeds (cv. Sids-19) were subjected to three plasma doses (exposure times of 0, 1.0, and 2.0 min). On 25th June, 2022 and 12th June, 2023, five cowpea seeds per hill with spacing of 20.0 cm were sown on two sides of the ridge in soils with low salinity (EC of 3.0 dS m^−1^), medium salinity (EC of 5.5 dS m^−1^), and high salinity (EC of 7.0 dS m^−1^). Plants were fertilized with 100 kg K_2_O ha^−1^ potassium sulfate (48% K_2_O), 35 DAS. Every, 12 day-interval, plants were irrigated through furrow flood irrigation system.

#### Agronomic assessments

Cowpea plants were harvested on 6th September 2022 and 25th August 2023. Plant samples were taken to measure plant height, number of branches per plant, number of leaves per plant, fresh weight of leaves per plant, and total forage yield per plant.

#### Agronomic data analysis

The experiments in wire house of in open field were structured in a randomized complete block design with four replicates. According to^41^, data from the field experiment were subjected to analysis of variance (ANOVA) using the Costat software program, Version 6.303 (2004). Means separation was performed only when the F-test indicated significant (*P* < 0.05) differences among the treatments, according to Duncan’s multiple-range test^42^.

### In vitro study

To assess the in vitro features of cowpea forage biomass, air-dried samples of forage yield were utilized. Chemical composition, degradability attributes, gas production attributes, and fermentation attributes were laboratory-estimated.

#### Chemical composition

Chemical analyses were conducted to estimate crude protein (CP), crude fiber (CF), ether extract (EE), and total ash according to the procedures of^43^. Neutral detergent fiber (NDF) and acid detergent fiber (ADF) were determined using the sequential procedures of^44^ using the Ankom200 (Ankom Technology Corp., Fairport, NY) filter bag technique. Additionally, organic matter (OM) was computed as 100 − total ash, and nitrogen-free extract (NFE) was calculated by difference, where NFE = OM − (CP + CF + EE). Gross energy (GE), digestible energy (DE), metabolizable energy (ME), and total digestible nutrients (TDN) were calculated according to^45^.

#### In vitro gas production

An in vitro batch culture technique was applied as described by^46^. Approximately 500 ± 3 mg of sample was weighed into 120 mL incubation vessels. At least three vessels were included as blanks, and alfalfa hay (H) and concentrate (CONC) were used in triplicate as standards to generate the correction factor. Rumen fluid was obtained from adult sheep aged 2 years, fed clover hay for 2 weeks immediately after slaughter at Al-Marg slaughterhouse. The collected rumen fluid was mixed and squeezed through four-layer cheesecloth into a 2 L bottle with an O_2_-free headspace and maintained in an insulated container containing warm water at 39 °C, then immediately transported to the laboratory.

Buffer solution was prepared according to McDougall: the buffer was made up of 9.8 g NaHCO_3_, 2.44 g Na_2_HPO_4_, 0.57 g KCl, 0.47 g NaCl, 0.12 g MgSO_4_·7H_2_O, and 0.16 g CaCl_2_·2H_2_O per liter of distilled water. It is important to note that CaCl_2_ must be added only after all the other components have completely dissolved. During the warming and reducing step, urea was added to the buffer at a rate of 1.0 g/L. Rumen fluid was mixed with the buffer solution in a ratio of 1:4 (v/v) to use as a source of inoculum. Each vessel was filled with 50 mL of the incubation medium and dispensed anaerobically before being closed. The samples were then incubated at 39 °C for 24 h. Finally, the vessels were randomly distributed in the rack in the incubator, and the tubes were swirled continuously.

Volumes of gas produced (GP) were measured after 24 h using a 100 mL glass syringe as described by^47^ and validated by^48^. To calculate the accurate volume of GP, Eq. (8) was used.

8 $$GP\,\left( {\text{ml/sample}} \right) = V_{24} {-}GP_{0} \times \left( {F_{H} + F_{CONC} } \right)/2,$$

where GP: The produced gas, V_24_: The volume of gas produced after 24 h of incubation, GP_0_: The volume of gas produced by the blank after 24 h of incubation, F_H_: The volume of gas produced by standard hay/recorded gas production of standard hay, F_CONC_: The volumes of gas produced by standard concentrate/recorded gas production of standard concentrate.

After 24 h of incubation, the gas production was recorded. Then, the filtration process was performed on each of the 120 mL vessels using a filter bag (F57 Ankom). After the filtration process, the filter bags were dried at 105 °C for 3 h in an oven to estimate residual dry matter (DM). After that, DM degradability (DMD), NDF degradability (NDFD), and ADF degradability (ADFD) were calculated as the difference between the weight of the incubated substrate and the weight of the non-degraded residue at the end of incubation, according to the Eq. (9)^44^

9 $${\text{IVD}}\;\left( \% \right) = (({\text{R}}{-}{\text{P}}))/{\text{R}} \times 100$$

where IDV: In vitro degradability, R: weight of the sample inside, P: the true weight of the out sample.

After 24 h of incubation, the gases produced and corrected by gases of the blank tubes were used to calculate gas production per g DM, OM, NDF, and ADF. The pH values, ammonia, and total volatile fatty acids (TVFs) concentrations were determined in the liquid part. The pH of rumen liquor was immediately recorded using a pH meter. Rumen liquor samples were analyzed to determine ammonia concentration (NH_3_) by Nessler’s method modified by^46^ and TVFs by steam distillation according to^49^.

#### In vitro data analysis

The in vitro data were analyzed as a factorial experiment (3 plasma doses × 3 salinity levels) in a completely randomized design, using the statistical analysis system SAS software^50^. Separation among means was carried out according to Duncan’s multiple-range test^42^ when the main factor was significant and at a confidence level of 95%. The collected data were subjected to the analysis of variance in two ways with the interaction. According to the General Linear Model, the statistical model used is illustrated in the Eq. (10).

10 $${\text{Y ijk}} = \mu + \upalpha {\text{I}} + \upbeta {\text{j}} + (\upalpha \upbeta ){\text{ IJ}} + {\text{E ijk}}$$

where Y ijk: the Kth Observation on the sample subjected to factors I and J, Μ: general average, α I: effect of the seed plasma exposure duration (i = 1, 2 and 3) I, βj: effect of soil salinity level (j = 1, 2 and 3), eij: residual error on the ruminal liquid subjected to factors I and J, (α β) I J: effect of the interaction between factors I and J.

## Results

### The efficient plasma dose

The plasma-treated cowpea seeds demonstrated significant modifications in surface chemistry and microstructure. Plasma exposure introduced oxygen- and nitrogen-containing functional groups (–OH, –COOH, –NOx), that may increase the surface energy and hydrophilicity, thereby enhancing water retention and nutrient absorption (Fig. 1 and Table 1). Controlled etching may alter the seed coat’s roughness, improving microbial interactions and gas exchange. The plasma dose response followed a biphasic hormesis curve, where moderate exposure (e.g., 2 min) optimized seed performance by enhancing germination and salinity tolerance.

**Fig. 2:**
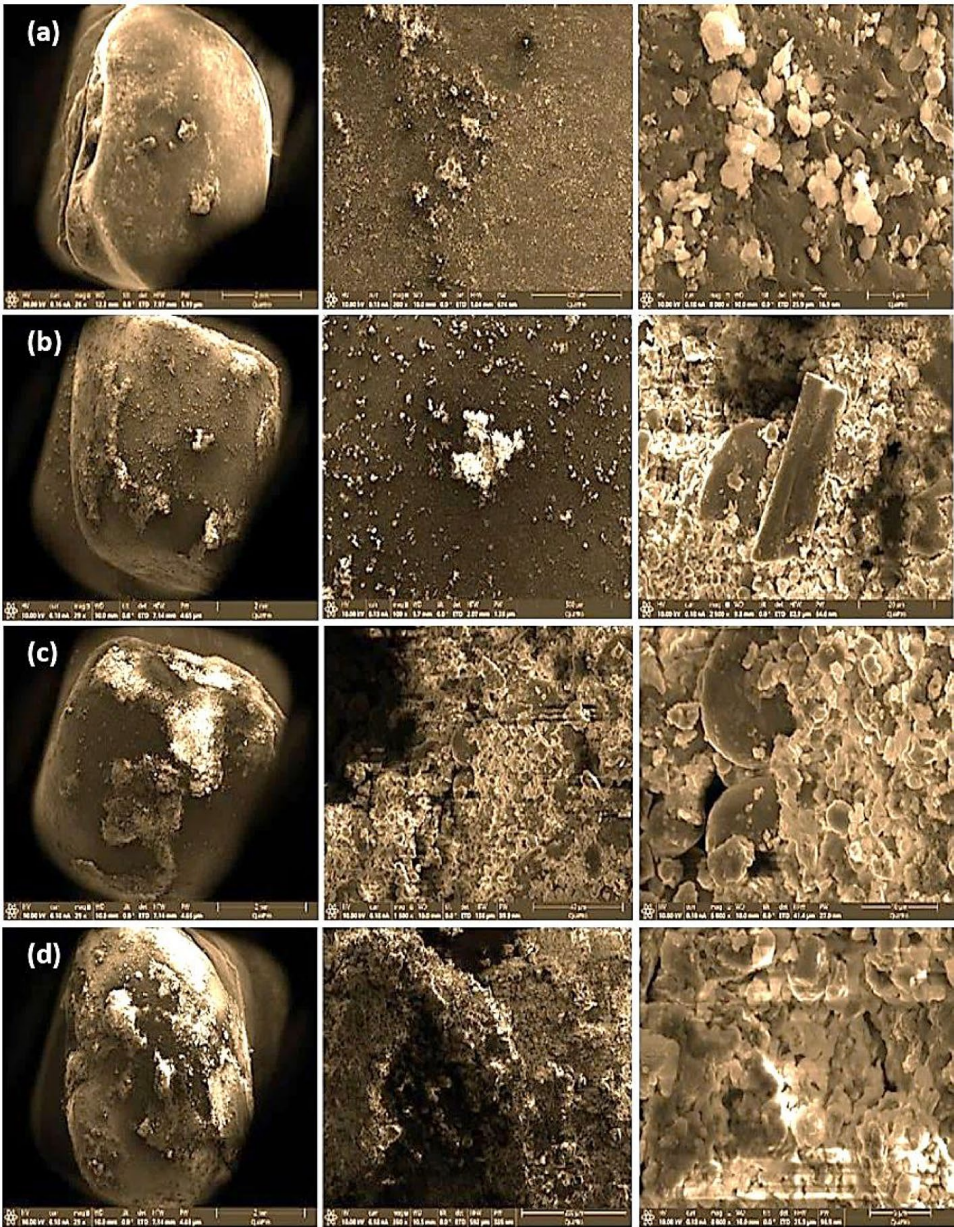
SEM micrographs of cowpea exposed to different plasma exposure times [(**a**) non-treated, (**b**,**c**) and (**d**) treating seeds with plasma for 1.0, 2.0 and 3.0-min, respectively]. The images are shown at different scales as indicated.

SEM images (Fig. 2) show a gradual increase in seed surface roughness with surface damage by prolonging plasma exposure time. The control treatment shows natural surface wrinkling; 1.0 min treatment introduces mild pitting; 2.0 min treatment results in deep cracking and topographical modulation; 3.0 min treatment causes exfoliation and carbonization dominate the surface. Furthermore, elemental detection composition (EDX) analysis revealed that when the plasma-treated seed sample was subjected to an electron beam, the emitted X-rays showed different responses at different exposure times (Table 2). In this respect, 2.0 min showed the best favourable effects, since calcium, phosphorus, magnesium molybdenum and organic integrity increased while sodium decreased. Despite 3.0 min showed the extreme calcium, sodium was also hugely increased with damaging the organic structure loss.

**Table 2:** Detcetion of the elemental composition analysis of cowpea plasma treated-seeds at different exposure times.

Element	0.0 min (Control)	1.0 min (Partial enhancing)	2.0 min (Optimal)	3.0 min (Risky)	Notes
Calcium (Ca%)	6.03	14.83 (↑146%)	19.32 (↑220%)	38.84 (↑544%)	Ca blocks Na⁺ influx and stabilizes membranes. 2-min = ideal uptake without damage
Sodium (Na%)	3.22	2.27 (↓30%)	1.92 (↓40%)	6.58 (↑104%)	Na⁺ buildup = toxicity. 3-min leaks Na⁺; 2-min = tight control
Phosphorus (P%)	0.70	1.74 (↑149%)	1.93 (↑176%)	2.71 (↑287%)	Key for ATP and root strength. Overexposure ≠ better; 2-min is efficient
Magnesium (Mg%)	1.31	3.59 (↑174%)	4.08 (↑211%)	4.85 (↑270%)	Mg supports chlorophyll and ionic balance. 2-min = high but safe
Molybdenum (Mo)	✖ Absent	✔ Detected	Present	Present	Essential for nitrogen metabolism. 2-min ensures trace element uptake
Organic content (C/O)	Stable	Slight drop	Stable	✖ Collapse	Organic integrity = seed viability. 3-min damages lipids/cell walls

### Cowpea growth and yield attributes

**Fig. 3:**
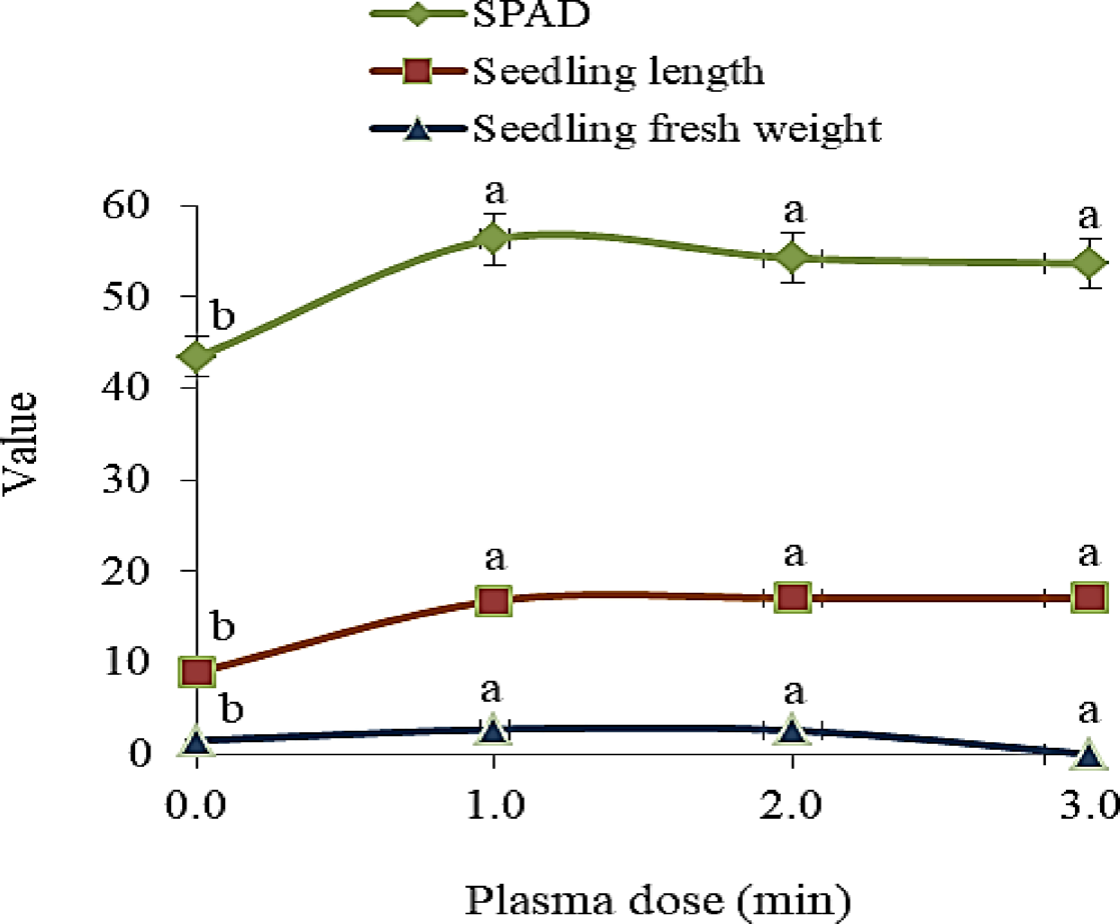
Relative chlorophyll content (SPAD), length (cm) and fresh weight (g) of cowpea seedling produced from plasma-treated seeds at different exposure times (0.0, 1.0 and 2.0-min).

**Fig. 4:**
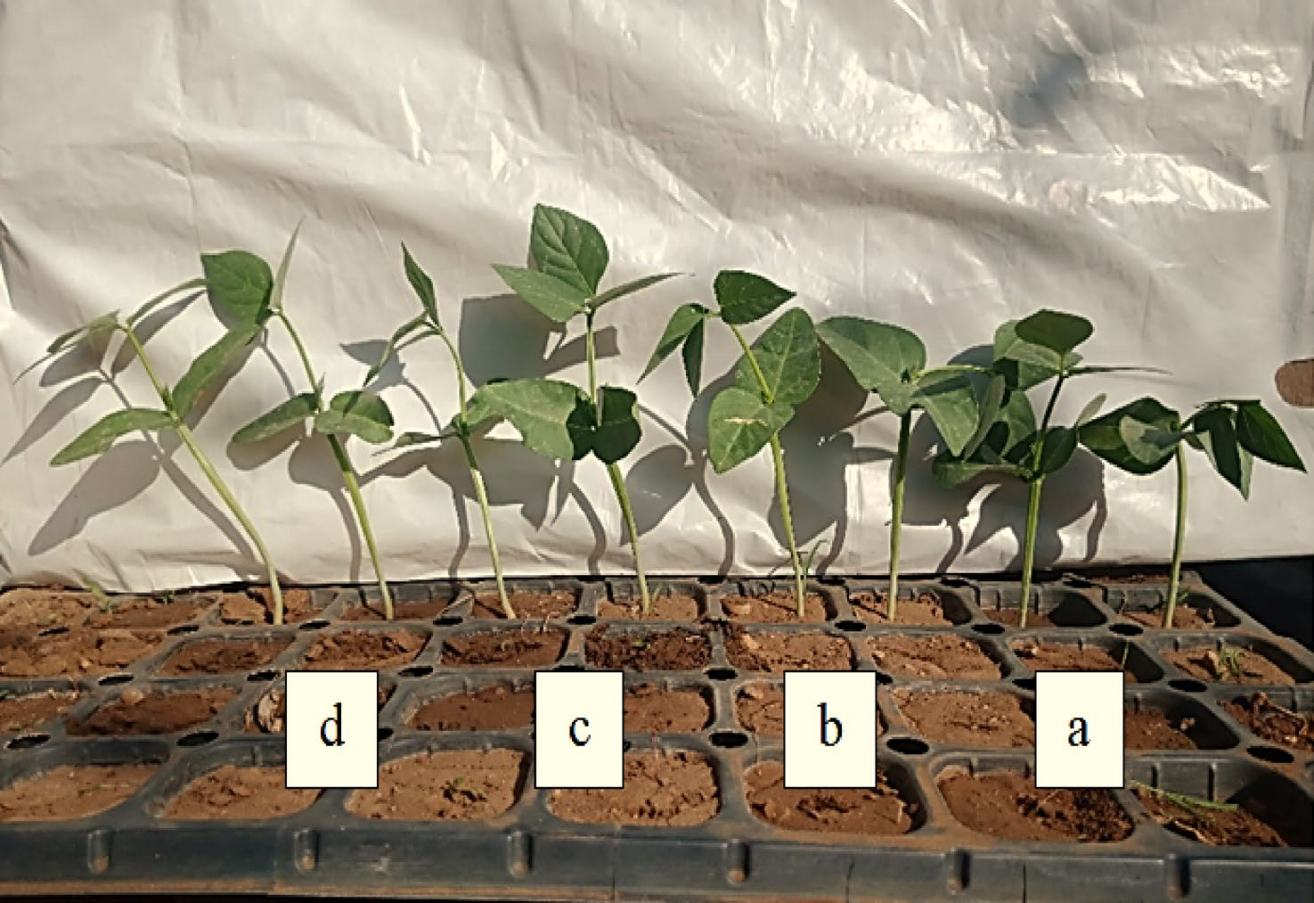
The growth attitudes of cowpea seedling produced from various plasma exposure times [(**a**) non-treated, (**b**–**d**) treating seeds with plasma for 1.0, 2.0 and 3.0-min, respectively].

Regarding the data of the pilot experiment in wire house, plasma treatment showed remarkable increases in all seedling growth characteristics of cowpea crop (Fig. 3). Seedlings produced form seeds that exposed to plasma for 1.0, 2.0 or 3.0 min surpassed the check treatment in SPAD value, seedling length and seedling fresh weight. With applications plasma for.0, 2.0 or 3.0 min, the increases amounted to 1.29, 1.24, 1.23 folds in relative chlorophyll content, 1.86, 1.91 folds in seedling length, and 1.90 and 1.80, 1.73 as well as 1.47 folds in seedling fresh weight, respectively, compared to the check treatment. The growth attitudes of cowpea seedling produced from various plasma exposure times are presented in Fig. 4.

As for open field study, data from the two seasons of 2022 and 2023 (Tables 3 and 4) showed that plasma treatment had positive effects on yield traits of cowpea cultivar Sids-19 under normal or saline conditions. Generally, exposing seeds to plasma for 1.0 min and 2.0 min treatments was effective for enhancing the yield and yield traits whether under normal or saline conditions as compared to the untreated seeds. However, the improvements in plant height due to plasma application were more evident under high salinity in the first season (with 1.0 min of plasma) and under all salinity levels in the second (with 1.0 min or 2.0 min of plasma) compared to the counterpart control treatment (0.0 min of plasma). Also, compared to the no plasma application (0.0 min) plasma treatment for 1.0 min or 2.0 min achieved more remarkable enhancements in branches number plant^−1^ under moderate salinity (in the second season) and severe salinity (in both seasons). With the application of plasma treatments, the increases in leaves number plant^−1^ and Leaves fresh weight plant^−1^ were more apparent under moderate salinity in the first season as well as under normal and the two salinity levels in the second season.

**Table 3:** Response of yield traits of cowpea cultivar Sids-19 to plasma exposure time under different salinity levels in 2022 growing season.

Variable	Plant height (cm)	Branches number plant^−1^	Leaves number plant^−1^	Leaves fresh weight plant^−1^ (g)	Total forage yield plant^−1^ (g)
EC3.0					
2.0 min	132.8ab	4.75a	43.5ab	67.4a–c	193.3ab
1.0 min	115.8ab	3.75ab	32.1a–d	53.4a–d	138.2b–d
0.0 min	129.4ab	4.33ab	40.1a–c	70.6ab	140.2b–d
EC5.5					
2.0 min	128.1ab	5.08a	40.5ab	80.4a	196.7ab
1.0 min	127.1ab	3.25ab	28.3a–e	48.9bc–e	147.6bc
0.0 min	116.4ab	3.00ab	22.3de	37.9c–e	109.7cd
EC7.0					
2.0 min	53.1cd	3.66ab	21.2de	36.9de	77.7de
1.0 min	67.1c	4.33ab	30.2a–e	53.8a–d	114.3cd
0.0 min	38.3d	2.58b	13.8e	20.2e	40.2e

EC3.0, EC5.5 and EC7.0: soil salinity at 3.0, 5.5 and 7.0 dS m^−1^, respectively; 0.0, 1.0, and 2.0 min: the plasma exposure times by minute. Different letters within columns refers that there are significant variations at 0.05 level of probability. Means were separated based on Duncan’s multiple range test (*P* ≤ 0.05).

**Table 4:** Response of yield traits of cowpea cultivar Sids-19 to plasma exposure time under different salinity levels in 2023 growing season.

Variable	Plant height (cm)	Branches number plant^−1^	Leaves number plant^−1^	Leaves fresh weight plant^−1^ (g)	Total forage yield plant^−1^ (g)
EC3.0					
2.0 min	123.8a	3.67ab	29.75a	48.58a	144.9a
1.0 min	112.7a–c	3.33ab	20.92a–c	45.92a	153.3a
0.0 min	94.87b–d	2.67a–c	14.42cd	23.00bc	90.00cd
EC5.5					
2.0 min	117.4ab	4.00a	24.83ab	36.17ab	131.3ab
1.0 min	101.9a–c	3.00a–c	17.67bc	26.58bc	101.0bc
0.0 min	86.20c–e	2.00bc	8.416d	16.08cd	51.77ef
EC7.0					
2.0 min	71.27de	3.33ab	19.67bc	23.67bc	54.87e
1.0 min	63.60ef	3.67ab	25.25ab	26.25bc	65.13de
0.0 min	42.97f	1.33c	8.250d	9.583d	20.60f

EC3.0, EC5.5 and EC7.0: soil salinity at 3.0, 5.5 and 7.0 dS m^−1^, respectively; 0.0, 1.0, and 2.0 min: the plasma exposure times by minute. Different letters within columns refers that there are significant variations at 0.05 level of probability. Means were separated based on Duncan’s multiple range test (*P* ≤ 0.05).

Compared to the corresponding check treatment (without plasma, 0.0 min), the exposure to plasma for 2.0 min in the first season was efficient for enhancing forage yield under normal (1.37-fold increase) and medium salinity (1.79-fold increase). While exposure to plasma for 1.0 min showed a better effect on forage yield under severe salinity. In the second season, both 1.0 min and 2.0 min of plasma surpassed the counterpart cheek plasma treatment (0.0 min) for improving forage yield.

### Cowpea in vitro features

#### Chemical composition

The data presented in Table 5 illustrates the effect of seed plasma exposure time and salinity levels on the chemical composition of cowpea plants. The data revealed that plasma treatment did not significantly affect ash, organic matter (OM), crude fiber (CF), crude protein (CP), neutral detergent fiber (NDF), and nitrogen-free extract (NFE) contents. Additionally, there were no significant differences (*P* > 0.05) in the calculated energy contents, including gross energy (GE), digestible energy (DE), metabolizable energy (ME), and total digestible nutrients (TDN) values. However, cowpea plants grown from seeds exposed to plasma for 1.0 min had significantly higher EE (*P* < 0.0001) compared to those exposed for 2.0 min and the untreated seeds (0.0 min), with no significant difference between the latter two treatments. Conversely, cowpea plants grown from seeds exposed to plasma for 1.0 and 2.0 min showed significantly higher acid detergent fiber (ADF) content (*P* = 0.0008) than those grown from untreated seeds with no significant differences between the two exposure times.

**Table 5:** Changes in chemical composition of cowpea cultivar Sids-19 in the in vitro systems (g/kg dry matter basis) due to plasma exposure time under different salinity levels.

Variable	0.0 min			1.0 min			2.0 min			*P* value		
	EC3.0	EC5.5	EC7.0	EC3.0	EC5.5	EC7.0	EC3.0	EC5.5	EC7.0	Plasma	Salinity	Interaction
Ash, g/kg	108	117	157	114	119	161	120	130	147	0.253	< 0.0001	0.046
Organic matter, g/kg	892	883	843	886	881	839	880	870	853	0.253	< 0.0001	0.046
Crude fiber, g/kg	306	340	231	281	263	248	295	349	261	0.14	0.009	0.274
Crude protein, g/kg	124	117	113	138	133	139	133	122	142	0.194	0.006	0.283
Ether extract, g/kg	28	19	24	21	26	32	24	27	22	< 0.0001	< 0.0001	< 0.0001
Nitrogen-free extract, g/kg	434	408	444	445	458	421	428	372	428	0.259	0.444	0.339
Neutral detergent fiber, g/kg	532	556	476	549	545	459	577	546	508	0.435	0.012	0.778
Acid detergent fiber, g/kg	269	303	218	301	315	264	365	317	250	0.0008	< 0.0001	0.009
Gross energy, kcal/kg DM	3706.47	3628.05	3493.38	3648.86	3648.86	3516.97	3639.48	3612.98	3519.97	0.335	< 0.0001	0.0559
Digestible energy, kcal/kg DM	2816.91	2757.32	2654.96	2773.13	2773.13	2672.89	2766.01	2745.86	2675.17	0.335	< 0.0001	0.0559
Metabolizable energy, kcal/kg DM	2309.87	2261	2177.07	2273.97	2273.97	2191.77	2268.12	2251.61	2193.64	0.335	< 0.0001	0.0559
Total digestible nutrients, %	63.89	62.54	60.21	62.897	62.89	60.62	62.73	62.27	60.67	0.335	< 0.0001	0.0559

EC3.0, EC5.5 and EC7.0: soil salinity at 3.0, 5.5 and 7.0 dS m^−1^, respectively; 0.0, 1.0, and 2.0 min: the plasma exposure times by minute.

Regarding the effect of soil salinity on the chemical composition of cowpea plants, the data of Table 5 show that ash content significantly gradually increased (*P* < 0.0001) as salinity levels increased from EC = 3.0 to EC = 7.0, with significant differences among the three salinity levels. Conversely, there was a significant decrease in OM content (*P* < 0.0001) with increasing salinity level from EC = 3.0 to EC = 7.0, with significant differences among the three salinity levels. Additionally, the plants grown at EC = 7.0 recorded significantly lower CF, NDF, and ADF (*P* = 0.009, *P* = 0.006, *P* = 0.012, and *P* < 0.0001, respectively) compared to those grown at EC = 3.0 and EC = 5.5, with no significant differences between the latter two treatment. Conversely, cowpea plants grown at the highest salinity level (EC = 7.0) recorded higher CP content (*P* = 0.006) compared to those grown at EC = 3.0 and EC = 5.5, with no significant differences between the latter two treatments.

Cowpea plants grown at EC = 7.0 recorded significantly higher EE content (*P* < 0.0001) compared to those grown at EC = 3.0, while the lowest EE content was recorded for those plants grown at EC = 5.5, showing significant differences among the three salinity levels. No significant differences were observed among the different salinity levels in NFE content (*P* = 0.444). Moreover, the energy values, including GE, DE, ME, and TDN, significantly decreased with increasing salinity levels (*P* < 0.0001), with significant differences among the three salinity levels.

The interaction between plasma treatment duration and salinity levels significantly affected ash, OM, EE, and ADF content (*P* < 0.05). The data showed that the plants grown from seeds treated with plasma for 2.0 min displayed lower ash content and higher OM content under salinity levels EC = 7.0 compared to the other treatments. The interaction effect on EE content (*P* < 0.0001) showed that plasma treatment applied for 1.0 min increased the EE content in cowpea plants grown under salinity levels at EC = 5.5 and EC = 7.0 compared to the other treatments. Concerning ADF content, plasma-treated seeds for 2.0 min displayed higher ADF content under salinity levels at EC = 3.0 and EC = 5.5 compared to the other treatments. In contrast, the interaction effect on cowpea feeding values such as GE, DE, ME, and TDN, was not significant and showed relatively minor variations across treatments.

#### Degradability attributes

The data presented in Table 6 illustrates the effects of seed plasma exposure time and salinity levels on the in vitro of dry matter degradability (DMD), neutral detergent fiber degradability (NDFD), and acid detergent fiber degradability (ADFD) in cowpea plants after 24 h of incubation. The results indicate that the in vitro DMD and NDFD in cowpea plants was not significantly influenced by seed plasma treatment (*P* = 0.167 and *P* = 0.8651, respectively). In contrast, plants grown from seeds exposed to plasma for 1.0 and 2.0 min showed significantly higher in vitro ADFD (*P* < 0.0001) compared to those from untreated seeds, with no significant difference between the two exposure times.

**Table 6:** Changes in degradability of dry matter (DMD), neutral detergent fiber (NDFD), and acid detergent fiber (ADFD) of cowpea cultivar Sids-19 in the in vitro systems after 24 h of in vitro incubation due to plasma exposure time under different salinity levels.

Variable	DMD	NDFD	ADFD
2.0 min			
EC3.0	68.58cb	59.44d	57.90a
EC5.5	66.24b–d	51.70de	62.13a
EC7.0	70.41b	60.89ab	71.26a
1.0 min			
EC3.0	60.71cd	45.28e	54.79a
EC5.5	66.59b–d	53.67d	62.73a
EC7.0	80.12a	65.63a	75.48a
0.0 min			
EC3.0	60.03d	40.12de	57.44a
EC5.5	65.02b–d	53.09cd	63.72a
EC7.0	71.08b	50.59bc	69.17a
SE	2.53	1.79	2.05
*P* value			
Plasma	0.167	< 0.0001	0.8651
Salinity	< 0.0001	< 0.0001	< 0.0001
Interaction	0.0221	< 0.0001	0.2027

EC3.0, EC5.5 and EC7.0: soil salinity at 3.0, 5.5 and 7.0 dS m^−1^, respectively; 0.0, 1.0, and 2.0 min: the plasma exposure times by minute.

Regarding salinity levels, the data revealed that cowpea plants grown at the highest salinity level (EC = 7.0) had significantly higher values of DMD, NDFD, and ADFD (*P* < 0.0001) compared to those grown at moderate and lower salinity levels (EC = 3.0 and EC = 5.5). Interestingly, plants grown at a moderate salinity level (EC = 5.5) had higher NDFD, and ADFD values compared to those grown at the lowest salinity level (EC = 3.0).

The interaction between seed plasma exposure time and salinity levels significantly affected DMD (*P* = 0.0221) and ADFD (*P* < 0.0001). Specifically, plants grown from seeds treated with plasma for 1.0 min showed higher DMD levels at the highest salinity level (EC = 7.0) compared to the other treatments. Furthermore, plants grown from seeds treated with plasma for both 1.0 and 2.0 min had higher ADFD values at high and moderate salinity levels (EC = 7.0 and EC = 5.5) compared to the other treatments. However, no significant interaction effect was noted for NDFD (*P* = 0.2027).

#### Gas production attributes

The presented data in Table 7 indicated that plasma treatment significantly affected all gas production parameters (*P* < 0.0001), including gas production per gram of dry matter (GP/g DM), organic matter (GP/g OM), neutral detergent fiber (GP/g NDF), and acid detergent fiber (GP/g ADF). Specifically, GP/g DM and GP/g OM were significantly lower (*P* < 0.0001) in cowpea plants grown from seeds exposed to plasma for 1.0 min compared to those from seeds treated for 2.0 min and untreated seeds. However, no significant differences were found between the plants from 2.0-min plasma-treated seeds and untreated seeds. Furthermore, the data showed that plants grown from untreated seeds exhibited significantly higher GP/g NDF and GP/g ADF (*P* < 0.0001) compared to those from seeds treated for 1.0 and 2.0 min. While there were no significant differences in GP/g NDF between plants grown from seeds treated with the two exposure times. GP/g ADF was significantly higher in plants grown from 2.0 min plasma-treated seeds than in those from 1.0-min plasma-treated seeds.

**Table 7:** Changes in gas production (GP) of dry matter (DM), organic matter (OM), neutral detergent fiber (NDF) and acid detergent fiber (ADF) of cowpea cultivar Sids-19 in the in vitro systems after 24 h of in vitro incubation due to plasma exposure time under different salinity levels.

Variable	GP, ml/g DM	GP, ml/g OM	GP, ml/g NDF	GP, ml/g ADF
2.0 min				
EC3.0	165.97bc	190.77cd	282.18ef	485.62ef
EC5.5	181.03ab	205.78bc	291.87c–e	461.04f
EC7.0	193.35a	226.80a	352.63ab	715.90b
1.0 min				
EC3.0	188.25a	213.75ab	319.01cd	551.97d
EC5.5	157.18cd	177.48d	266.31f	485.56ef
EC7.0	147.71d	176.05d	306.51c–e	533.28ed
0.0 min				
EC3.0	181.24ab	205.19bc	300.67cde	550.80d
EC5.5	187.78a	210.55ab	327.18bc	646.62c
EC7.0	183.04a	217.11ab	357.96a	781.07a
SE	5.13	5.98	9.64	17.19
*P* value				
Plasma	< 0.0001	< 0.0001	0.001	< 0.0001
Salinity	0.63	0.2099	< 0.0001	< 0.0001
Interaction	< 0.0001	< 0.0001	0.0002	< 0.0001

EC3.0, EC5.5 and EC7.0: soil salinity at 3.0, 5.5 and 7.0 dS m^−1^, respectively; 0.0, 1.0, and 2.0 min: the plasma exposure times by minute.

Regarding the effect of salinity on gas production parameters, no significant differences in GP/g DM and GP/g OM were observed with different salinity levels (*P* = 0.63 and *P* = 0.2099, respectively). However, plants grown under high salinity conditions (EC = 7.0) displayed significantly higher GP/g NDF and GP/g ADF (*P* < 0.0001) compared to those grown at moderate (EC = 5.5) and low salinity levels (EC = 3), with no significant differences between the moderate and low salinity levels.

The interaction between plasma treatment and salinity was highly significant (*P* < 0.0001) across all GP parameters, highlighting the complex relationship between these two factors.

#### Fermentation attributes

The data presented in Table 8 showed the effects of seed plasma exposure time and salinity levels on in vitro fermentation parameters, including pH value, ammonia (NH_3_), and total volatile fatty acids (TVFAs). The data indicated that plants grown from seeds treated with plasma for 1.0 min had significantly higher ammonia concentrations (*P* < 0.0001) compared to those grown from seeds treated for 2.0 min and untreated seeds, which showed no significant difference among them. In contrast, plants grown from seeds treated with plasma for 2.0 min showed significantly higher TVFAs concentrations (*P* = 0.0327) than those from seeds treated for 1.0 min and the untreated seeds, with no significant difference observed between the latter two treatments. However, plasma exposure did not significantly affect pH values (*P* = 0.3845).

**Table 8:** Changes in fermentation attributes of cowpea cultivar Sids-19 in the in vitro systems after 24 h of in vitro incubation due to plasma exposure time under different salinity levels.

Variable	pH value	Ammonia, mg/dl	Total volatile fatty acids, mequ/dl
2.0 min			
EC3.0	6.65a	9.43ab	8.87a
EC5.5	6.63a	7.98bc	8.73a
EC7.0	6.58a	7.59c	9.15a
1.0 min			
EC3.0	6.60a	8.86b	7.28c
EC5.5	6.58a	9.5ab	9.10a
EC7.0	6.60a	9.91a	8.55ab
0.0 min			
EC3.0	6.52a	7.12c	8.65a
EC5.5	6.55a	6.43d	8.45ab
EC7.0	6.62a	10.70a	7.6bc
SE	0.05	0.31	0.33
*P* value			
Plasma	0.3845	< 0.0001	0.0327
Salinity	0.958	< 0.0001	0.1997
Interaction	0.6268	< 0.0001	0.0036

EC3.0, EC5.5 and EC7.0: soil salinity at 3.0, 5.5 and 7.0 dS m^−1^, respectively; 0.0, 1.0, and 2.0 min: the plasma exposure times by minute.

Plants grown under high salinity levels (EC = 7.0) recorded significantly higher (*P* < 0.0001) ammonia concentrations compared to those plants grown under moderate and low salinity conditions. However, salinity level had no significant effect on TVFAs production (*P* = 0.1997) or pH (*P* = 0.958).

A significant interaction effect between plasma treatment and salinity was observed for ammonia concentration (*P* < 0.0001) and TVFAs production (*P* = 0.0036), while no significant effect was recorded for pH value (*P* = 0.6268). The highest ammonia concentration was recorded in plants grown under EC = 7.0 and treated with plasma for 1.0 min, while the lowest was observed in 2.0-min plasma-treated seeds grown under EC = 7.0. In terms of TVFAs production, the interaction effect showed that 2.0-min plasma-treated seeds produced the highest TVFAs levels across different salinity conditions, particularly at EC = 7.0.

## Discussion

### Plasma dose and cowpea growth

All plasma-treated seeds exhibited improved agronomic performance for cowpea compared to the control. 2.0 min of exposure consistently provided the most balanced and effective enhancement. Along with the favorable modification in seed surface (as SEM proved), EDX analysis strongly suggests that the 2.0 min treatment enhances elemental nutrients (Table 2). However, it’s not conclusive unless backed by germination and seedling vigor assessments as well as growth/yield measurements in saline soil. In this context, the 2.0 min exposure time of seeds to plasma optimized surface activation, stress priming and mild etching, leading to superior water and nutrient uptake, and stress-response activation^8^. Despite 1.0 min of plasma exposure also yielded positive effects, 2.0 min struck the best balance. These results confirm that 2.0-min plasma treatment is the most effective dose for maximizing seed performance of cowpea especially under saline conditions. However, plasma exposure time could differ among different plant species. It has been observed increases of nitrogen complex in the seed coat up-regulating the protein via conversion of nitrogen that was responsible for improved seed germination, chlorophyll content, total soluble and sugar protein, and seedling growth grown from plasma-treated seeds for 1.5 min in black gram^13^. While, Rashid et al.^51^ stated that the stimulation effects of plasma on paddy seed germination rate, enzymatic activities, total soluble protein and sugar and eventually economic yield were more evident with plasma-treated seeds for 2.0 min.

### Cowpea productivity

The enhancement of seed germination and seedling growth is crucial for ensuring acceptable productivity of crops under saline soil conditions. By assessment the efficiency of plasma on cowpea seedling growth, it was observed that plasma at various exposure times showed noticeable enhancements in SPAD, seedling length, and seedling fresh weight compared to the control treatment (Fig. 3). Because of its etching effect and chemo-functionalization, plasma physically caused modification in seed surface to be more hydrophilic and permeable^52^, hence seedling growth behavior ameliorated. In this respect, NTP has shown substantial benefits in crop production, as it improves seed germination^53,54^ and resilience to abiotic stress^55^. However, little information is available related to the ability of NTP as an alleviator of salt stress. The current study attempted to highlight the relationship between exposure times of NTP on cowpea seeds sown in saline soils. The favorable effect of plasma on the growth and forage yield of cowpea was more evident under low (3.0 dS m^−1^) and moderate (5.5 dS m^−1^) salinity. This could be attributed to plasma having a protective role against the toxicity of salts via increased activity of peroxidase and phenylalanine ammonia lyase^56^. Further, the synthesis of plant hormones in seeds and seedlings was influenced by plasma treatments^57,58^.

After NTP application, gibberellin concentration and hydrolytic enzyme activity elevated, stimulating seed germination^59^. Up-regulation in cytokinin and auxins was observed after treating seeds with NTP^60^. Also, an increase in abscisic acid was obtained in wheat seedlings produced from NTP-treated seeds^61^. Abscisic acid is known to be an important key indicator in drought response, regulating stomatal conductance and water status while stimulating genes associated with drought resistance.

It has been documented that salts cause lipid peroxidation and cell membrane damage, expressed in the elevation of malondialdehyde (MDA)^62^. As an interesting performance, NTP treatment reduced MDA levels while enhancing germination and seedling growth, suggesting that timely exposure of seeds to plasma may maintain membrane integrity via quickening the antioxidant mechanism^17,55^. It has recently been reported that seeds exposed to plasma showed a high germination rate with stimulation of seedling growth and enhanced phytohormones, enzymatic activities, and stress resistance with improved productivity^9,63^. Although the cowpea forage yield attributes were improved using NTP-treated seeds under normal or mild salt stress conditions, further investigations are needed to explore the physiological and molecular changes in the adult plant beside the seedlings.

### In vitro attributes

#### Chemical composition

The insignificant differences (*P* > 0.05*P* > 0.05) in chemical analysis parameters such as ash, OM, CF, CP, NDF, GE, DE, ME, and TDN due to the period of seed plasma exposure suggest that this treatment had no adverse effects on the chemical composition of the grown cowpea plants. These findings support the potential use of seed plasma treatment as a strategy to enhance cowpea productivity in saline conditions. However, the highest recorded EE content in cowpea plants grown from seeds treated for 1.0 min, compared to those treated for 2.0 min and the untreated seeds, suggests that a 1.0-min seed plasma exposure may contribute to enhanced lipid accumulation.

Additionally, ADF content was significantly lower in plants derived from untreated seeds compared to those exposed to plasma for 1.0 or 2.0 min. Given that plant cell walls dynamically respond to abiotic stressors such as plasma exposure, this reduction may be linked to enzymatic activities that regulate cell wall remodeling. Enzymes such as xyloglucan endotransglucosylase/hydrolase (XTH) and expansion’s play crucial roles in maintaining cell wall plasticity under stress conditions^64^. This observation aligns with previous findings suggesting that plasma treatment can enhance carbohydrate metabolism, thereby improving stress tolerance mechanisms^52^.

Furthermore, the significant gradual increase in ash content alongside a significant gradual decrease in OM content with escalating salinity levels (from EC = 3.0 to EC = 7.0) suggests a potential reduction in carbohydrate and protein synthesis. This decline in organic matter at higher salinity levels aligns with studies indicating that salinity-induced oxidative stress can compromise the stability of organic compounds^65^. The observed significant decrease in CF, NDF, and ADF content with increasing salinity levels suggests possible inhibition of fiber biosynthesis and nitrogen assimilation due to salt stress. Ashraf and Harris^66^ attributed this decline to impaired protein synthesis and nitrogen metabolism under salt stress. Both NDF and ADF contents were reduced under higher salinity, indicating alterations in cell wall composition, consistent with findings by^64^. Moreover, the significant reduction in energy values (GE, DE, ME, and TDN) with increasing salinity levels reflects an overall decline in the nutritional quality of cowpea under saline conditions. These results may be attributed to a general decrease in organic matter and other nutrients, particularly nitrogen-free extract (NFE). The recorded non-significant effects on cowpea feeding values (GE, DE, ME, and TDN) suggest that plasma treatment may partially alleviate the reductions in energy values caused by salinity. Overall, these findings indicate that, while plasma treatment alone had limited influence on most parameters, the combination of plasma treatment with varying salinity levels significantly altered the chemical composition of cultivated cowpea plants, particularly regarding lipid accumulation and fiber structure.

#### Degradability

The data indicated that the in vitro dry matter degradability (DMD) and neutral detergent fiber degradability (NDFD) in cowpea plants were not significantly affected by seed plasma treatment, indicating that plasma treatment had no adverse effects on DMD and NDFD. However, a significant improvement in in vitro acid detergent fiber degradability (ADFD) was observed for cowpea plants grown from plasma-treated seeds compared to untreated seeds. This result may be due to cowpea seed plasma exposure inducing slight modifications in the cell wall structure and composition. These findings align with previous findings by^52,64^, which reported that abiotic stressors, including plasma exposure, can influence cell wall dynamics. The observed increase in ADFD corresponded with a rise in cellulose content, an easily fermentable substrate, further supporting this hypothesis.

The significant increase in DMD observed in plants grown under high salinity conditions (EC = 7.0) may be attributed to their higher ash content compared to those cultivated at moderate (EC = 5.5) and low (EC = 3.0) salinity levels. Since ash consists of soluble components, its elevated concentration could have contributed to the enhanced dry matter degradability. Additionally, the increase in in vitro NDFD and ADFD in plants grown at high salinity levels (EC = 7.0) may be attributed to several physiological and biochemical responses to salinity stress. Salinity can induce osmotic stress, leading to the accumulation of osmolytes such as soluble sugars and proteins, which help maintain cellular turgor and protect cellular structures. These osmolytes can enhance microbial activity during in vitro incubation, potentially increasing the degradability of plant tissues. A study by^67^ reported that cowpea plants exposed to salinity exhibited increased levels of soluble sugars and proteins, which could contribute to improving in vitro degradability. Moreover, salinity stress can alter the structural composition of plant cell walls, potentially reducing lignin content and modifying the proportion of structural carbohydrates. These changes can increase the susceptibility of cell walls to microbial breakdown, facilitating degradation. While specific studies on cowpeas are limited, research on other legumes suggests that salinity can modify cell wall composition, affecting degradability. Therefore, the highest degradability observed at EC = 7.0 may result from a combination of osmolyte accumulation and cell wall modifications enhancing microbial digestion efficiency.

The significant interaction effect observed for DMD suggests that the impact of plasma treatment varied across different salinity levels. The most recorded improvement in DMD due to plasma exposure was observed at high salinity levels (EC = 7.0), where degradability increased from 71.08% in cowpea plants grown from untreated seeds to 80.12% in plants grown from seeds treated for 1.0 min. Furthermore, the interaction effect on ADFD % was more pronounced under high salinity, where plasma treatment had the greatest impact at EC = 7.0, resulting in 65.63% ADFD for plants grown from seeds treated for 1 min and 60.89% for those treated for 2 min. At EC = 5.5, the plants grown from seeds treated for 2 min showed a significant increase in ADFD, raising it from 40.12% (untreated) to 59.44%. These findings indicate that the beneficial effects of plasma treatment on degradability are influenced by salinity levels, with more pronounced improvements occurring under higher salinity conditions.

#### Gas production

In vitro gas production is regarded as an indirect measurement of rumen fermentation, making it a useful indicator for quantifying nutrient utilization^68^. Although plasma exposure duration had no significant effect on dry matter (DM) and neutral detergent fiber (NDF) degradation (DMD and NDFD), the data showed a significant decrease in gas production per gram of dry matter (GP/g DM) and gas production per gram of organic matter (GP/g OM) in cowpea plants grown from seeds exposed to plasma for 1.0 min compared to those grown from seeds treated for 2.0 min and untreated seeds. This result suggests that prolonged plasma exposure (2.0 min) may mitigate the initial reduction observed with shorter treatment durations (1.0 min).

Concerning the superiority of gas production per gram of neutral detergent fiber (GP/g NDF) and gas production per gram of acid detergent fiber (GP/g ADF) for plants grown from seeds exposed to plasma for 2.0 min compared to other treatments, this may be due to the recorded higher fermentation for plants grown from seeds exposed to plasma for 2.0 min, as indicated by the higher total volatile fatty acids (TVFAs) concentration.

Although the plants grown at high salinity levels (EC = 7.0) recorded significantly higher DM degradability, the gas production per gram of DM and per gram of OM showed a non-significant effect, which can be explained by the increased ash content in the plants grown at EC = 7.0. Ash consists of soluble components that disappear when determining degradability, thereby increasing the results.

However, the recorded significantly higher GP/g NDF and GP/g ADF for plants grown at high salinity levels (EC = 7.0) compared to those grown at moderate and low salinity levels (EC = 3.0 and EC = 5.5) could be attributed to the recorded significant.

#### Fermentation

The observed increase in ammonia concentration under high salinity conditions indicates a change in nitrogen metabolism, likely resulting from osmotic stress impacting microbial activity. However, total volatile fatty acids (TVFAs) production was significantly enhanced in plants grown from seeds treated for 2.0 min, suggesting improved fermentation efficiency. These findings align with previous studies showing that plasma treatment can influence secondary metabolite pathways^65^, and also agree with the hypothesis that plasma treatment enhances carbohydrate metabolism, promoting stress tolerance mechanisms^52^ and indirectly affecting fermentation dynamics. However, plasma exposure did not significantly affect pH values, indicating that the plasma treatment did not alter the overall acidity of the fermentation environment.

Concerning the effect of salinity levels, the data showed that the plants grown under high salinity levels (EC = 7.0) recorded significantly higher ammonia concentration compared to those grown at moderate and low salinity (EC = 3.0 and EC = 5.5). This increase in ammonia concentration could be attributed to a disruption in nitrogen metabolism under salt stress, as noted by^65^. However, the salinity level had no significant effect on TVFAs production or pH value, suggesting that ruminal fermentation activity remained stable across different salinity conditions. Previous studies^46^ have reported similar findings, indicating that while salinity affects nitrogen metabolism, microbial fermentation efficiency can remain largely unaffected under moderate stress conditions.

The significant interaction effect due to plasma treatment and salinity level suggests that plasma treatment modifies fermentation responses under saline conditions, with 2.0-min plasma exposure being the most effective in optimizing ammonia and TVFAs balance in cowpea plants subjected to different salinity levels.

## Conclusions

It could be summarize that plasma as safe seed priming can be exploited to sustain cowpea productivity in saline soil. Herein, the current study exhibited that subjecting cowpea seeds for 2.0 min before sowing led to good establishment and yield under salty soil. The findings also highlighted the potential of plasma treatment in optimizing feed quality under different salinity conditions, with 2-min plasma exposure showing the most pronounced benefits at high salinity levels. The integration of plasma technology into modern agricultural practices could revolutionize crop production, particularly in regions affected by soil salinity and climate-induced stressors. By preparing seeds with the ability to survive saline environments, this method contributes to improving global food security, especially in areas where traditional farming struggles with salt accumulation. However, further future investigations are needed to estimate the potential role of plasma on various plant species and varieties at different exposures times while assessing the possible physiological, biochemical and molecular modifications.

## Data Availability

All data supporting the findings of this study are available within the paper.

## Abbreviations

EC: Electrical conductivity
NTP: Non-thermal plasma
CP: Crude protein
CF: Crude fiber
EE: Ether extract
NDF: Neutral detergent fiber
ADF: Acid detergent fiber
OM: Organic matter
DM: Dry matter
GE: Gross energy
DE: Digestible energy
ME: Metabolizable energy
TDN: Total digestible nutrients
DMD: Dry matter degradability
NDFD: Neutral detergent fiber degradability
ADFD: And acid detergent fiber degradability
TVFs: Total volatile fatty acids
